# Dataset on farmers’ perception of commodity futures market

**DOI:** 10.1016/j.dib.2022.108429

**Published:** 2022-06-30

**Authors:** S. Srinivasan, M. Babu, P.S. Shabi Shimny, C. Hariharan, J. Gayathri, G. Indhumathi

**Affiliations:** a Faculty of Management Sciences, Sri Ramachandra Institute of Higher Education and Research, Chennai, Tamil Nadu, India; b Bharathidasan School of Management, Bharathidasan University, Tiruchirappalli, TamilNadu, India; c Gulf Centre for University Education (IGNOU), Shuwaikh 70455, Kuwait; d Department of Management Studies, Nehru Institute of Technology, Coimbatore, Tamil Nadu, India; e Department of Commerce and Financial Studies, Bharathidasan University, Tiruchirappalli, TamilNadu, India; f Department of Commerce, Mother Teresa Women's University, Kodaikanal, India

**Keywords:** Price risk hedging, Interview, Data cleaning

## Abstract

The commodity futures market plays a major role in reducing the price risk for the participants. Unfortunately, the farmers’ participation in the futures market particularly from the Tamil Nadu region is very less. A survey was conducted using the interview method to identify the information sources used by farmers for taking pricing decisions, the awareness and perception of farmers towards the futures market, and its effect on preferred marketing alternatives. The data cleaning process was done using content validity, confirmatory factor analysis, and reliability test using Cronbach's alpha, and the assumptions of normality and multicollinearity were examined. The data will be of potential use to researchers who wish to explore farmers’ behavior towards hedging in the commodity futures market.

## Specifications Table

**Table:** 

Subject	Agricultural Economics
Specific subject area	Financial Markets and Institutions
Type of data	Table
How the data were acquired	Interview
Data format	Raw Data- Ms Excel.
Description of data collection	The data captures details on demographic, farming, training, marketing, the awareness, and perception of the futures market from 314 chilly farmers, 383 Turmeric farmers, and 221 Cardamom growers in the state of Tamil Nadu, India. The following are the main inclusion criteria for the selection of respondents: (a) The participants must have cultivated either chilly, cardamom or Turmeric. Non-cultivators of chilly, turmeric or cardamom forms the exclusion criteria (b) The second inclusion criteria are the respondents cultivating the spices in the top three villages in the respective taluk by the area of cultivation 2015–16 statistics. The respondents who are growing the spices in other villages did not form part of the group. To identify the sample frame, a stage-wise split of the population was done from District to Taluks to Villages. The major challenge in identifying the respondents was village-wise data availability of farmers and the area cultivated by each farmer. The accessibility and retrieval of data from the taluk office was really a very difficult task. Hence, based on the inclusion and exclusion criteria and applying simple random sampling in the top three villages, the farmers were selected randomly from the three villages with the help of village administrators. The respondents were contacted individually and the required data were collected.
Data source location	***Institution****:* Bharathidasan University***City/Town/Region:*** Tiruchirappalli, Tamil Nadu***Country:*** India
Data accessibility	**Repository name:** Mendeley Data**Data identification number:** 10.17632/pw339snbvs.2**Direct URL to Data:**https://data.mendeley.com/datasets/pw339snbvs/2

## Value of the Data

• The data on Tamil Nadu farmers’ participation in futures markets is unknown through any of the sources.
• It will answer the following questions: How many farmers are using the futures market? What other marketing tools are used by farmers to sell their produce?
• This data will provide useful insights into farmers’ understanding of various aspects of commodity futures trading.
• This data will add value to the policymakers in developing strategic measures for protecting the farmers from price risk and exploitation by the traders.
• The researchers can make use of the data to develop models on farmers’ hedging behavior in the futures market.

## 1 Data Description

The interview schedule includes demographic data which were recorded on a nominal scale, training and farming details noted on an ordinal scale, the cost details were collected as open-ended, the preference of marketing alternatives recorded on a three-point scale with “1” Not preferred, “2” Preferred and “3” Highly preferred. The farmers’ awareness level of the futures market was collected using a Likert scale which includes “1” completely unaware and “5” being completely aware. Similarly, the perception of farmers towards the futures market was obtained using Likert scale responses which include “1” Strongly disagree and “5” Strongly agree. The data collection instrument included one section on the frequency of usage of information sources for taking pricing decisions which were recorded as “1” Never and “5” Always. The results of the reliability test are presented in Table 1, confirmatory factor analysis (CFA) for awareness, perception level of farmers towards futures market, and information sources used by farmers for taking pricing decisions are presented in Tables 2, 3, and 4, respectively. The assessment of normality of the data using the Kolmogorov Smirnov test is presented in Table 5 and the examination of multicollinearity in the data set was assessed using the Tolerance and Variance Inflation Factor which is presented in Table 6.

**Table 1:** Results of reliability test.

Constructs	Cronbach's Alpha Value
Preferred Marketing Alternatives	0.739
Information Sources used for taking Pricing decisions	0.775
Awareness level towards Futures Market	0.822
Perception level towards Futures Market	0.943
Selection of Marketing Alternatives	0.794

**Table 2:** Confirmatory factor analysis for awareness level towards futures.

	CR	AVE	MSV	ASV	CF	KP	TS
**CF**	0.9	0.566	0.187	0.097	0.752		
**KP**	0.874	0.635	0.006	0.003	−0.08	0.797	
**TS**	0.911	0.6	0.187	0.094	0.433	−0.016	0.775

**Note:** CF- Knowledge of Commodity Futures; KP-Knowledge about Price; TS- Knowledge of Trading & Settlement; CR-Critical Ratio; AVE- Average Variance Explained MSV-Maximum Shared Variance; ASV-Average Shared Variance.

**Table 3:** Confirmatory factor analysis for perception level of farmers towards futures market.

	CR	AVE	MSV	ASV	EP	EF	PRE	RA
**EP**	0.946	0.779	0.545	0.369	0.882			
**EF**	0.901	0.646	0.563	0.457	0.738	0.804		
**PRE**	0.878	0.592	0.563	0.378	0.55	0.75	0.769	
**RA**	0.804	0.531	0.269	0.264	0.51	0.513	0.519	0.729

**Note:** EP- Expected Performance from Futures; EF-Entrepreneurial Freedom; PRE-Perceived Risk Exposure from Futures; RA-Risk Attitude; CR-Critical Ratio/Composite Reliability; AVE- Average Variance Explained; MSV-Maximum Shared Variance; ASV-Average Shared Variance.

**Table 4:** Confirmatory factor analysis for information sources used by farmers for taking pricing decisions and preference of farmers towards selection of marketing alternatives.

	CR	AVE	MSV	ASV	MKT	IS
**MKT**	0.870	0.513	0.040	0.029	0.716	
**IS**	0.893	0.808	0.227	0.133	−0.200	0.899

**Note:** MKT- Marketing Alternatives; IS- Information Sources.

**Table 5:** Kolmogorov- Smirnov test of normality.

Factors	Information Sources	Awareness Level	Perception Level	Marketing Alternatives
n	918	918	918	918
Kolmogorov- Smirnov Z	1.09	1.06	1.05	0.95
Asymp.Sig. (2-tailed)	0.17	0.21	0.22	0.33

**Table 6:** Tolerance and Variance Inflation Factor for detecting multicollinearity.

Coefficients		
	Collinearity Statistics	
Model	Tolerance	VIF
Awareness	.843	1.186
Perception	.918	1.089
Information Sources used	.914	1.094

a. Dependent Variable: Selection of Marketing Alternatives.

The alpha coefficients for Preferred Marketing Alternatives, Information Sources, used by farmers for taking pricing decisions, the Awareness level of farmers towards futures market, Perception level of farmers towards futures market [1], and Selection of Marketing Alternatives were found to be 0.739, 0.775, 0.822, 0.943 and 0.794 respectively, suggesting that these items recorded relatively high internal consistency and hence the instrument was fit to collect data [2].

The results of confirmatory factor analysis are presented in Table 2, Table 3, Table 4. It is clear from Table 2 that the Critical Ratio (CR) was found to be 0.90, 0.874, and 0.911 for the constructs related to the awareness level of farmers towards the futures market, which includes Knowledge of Commodity Futures, Knowledge about Price, and Knowledge of Trading and Settlement. Similarly, the perception is measured through four constructs namely Expected Performance from futures, Entrepreneurial Freedom, Perceived Risk Exposure from futures, and the Risk Attitude [3], [4], [5], [6]. Table 3 presents the CR values for the same which includes 0.946, 0.901, 0.878, 0.804, respectively. The marketing alternatives and information sources recorded CR values of 0.870 and 0.893, respectively. Since all the CR values were greater than 0.7, the constructs given in the model were valid. The AVE values for Knowledge on Commodity Futures, Knowledge on Price, Trading and Settlement were found to be 0.566, 0.635, and 0.6 respectively which met the required standard. The Convergent Validity was measured, through Average Variance Extracted (AVE), which should be greater than 0.5. The Average Variance Explained (AVE) was found to be 0.779, 0.646, 0.592, and 0.531 respectively, for Expected Performance from Futures, Entrepreneurial Freedom, Perceived Risk Exposure, and Risk Attitude of farmers respectively and this ensures that constructs were convergent valid. It is clear from Table 4 that the AVE value for Marketing Alternatives, and for the Information Sources used by farmers for taking pricing decisions, was found to be 0.513 and 0.808, respectively. The results of Divergent/Discriminant Validity were evaluated, using diagonal values of each construct, which should be greater than AVE values. From the results, the Maximum Shared Variance (MSV) and Average Shared Variance (ASV) were less than the Average Variance Explained (AVE) and hence the factors are distinct and uncorrelated. The item loading, for all the constructs under awareness, perception, information sources used, and marketing alternatives was greater than 0.5, hence all the items perfectly fit the constructs [2].

It is to be noted that the Kolmogorov-Smirnov ‘Z’ Statistic from Table 5 was found to be 1.09, 1.06, 1.05, and 0.95, for Information sources used by farmers for taking pricing decisions, the Awareness level of farmers towards futures market, Perception level of farmers towards futures market and the Preference of farmers in the selection of Marketing Alternatives respectively. The *p*-value of Kolmogorov Smirnov ‘Z’ Statistic, was found to be greater than 0.05, indicating statistically insignificant results. Hence accept the null hypothesis, “The data follows normal distribution”.

The Variance Inflation Factor of independent variables, namely, the awareness and perception level of farmers towards futures market, the information sources used by farmers for taking pricing decisions were found to be 1.186, 1.089, and 1.094, respectively from Table 6. The VIF specifically gives the variances that are inflated in number which arises due to multicollinearity. The values, extracted in the model used in the research were well within the limit specified i.e. three, which indicates the absence of multicollinearity. Similarly, another indicator of multicollinearity is the Tolerance level. Generally, a higher level of tolerance is preferred as lower levels of tolerance can have adverse effects on the model. The Tolerance level, extracted for the present model, was found to be 0.843, 0.918, and 0.914 for awareness, perception level of farmers towards futures, and information sources used by farmers for taking pricing decisions respectively. These results confirmed that there was no multicollinearity in the data.

## 2 Experimental Design, Materials and Methods

Initially, it was decided to use the questionnaire method for collecting the data. During the pilot study, the farmers were contacted individually at the regulated markets where auctioning of turmeric, cardamom, and chilly are conducted. The informal discussions, with farmers’, revealed that the majority were not able to read and understand the questions. Some of the choices, with respect to information sources used by farmers, did not find a place in the questionnaire. All the above issues were addressed after the first pilot study. During the second pilot study, with 125 farmers’, additional marketing alternatives namely selling to the traders, co-operative societies, etc., were added. It was noticed during the interaction, that the farmers were not aware of different marketing alternatives for selling their produce. The only price risk instrument known to them was crop insurance and moreover, majority of the farmers were not enrolled in the crop insurance schemes. The outcome of the pilot study revealed the following: (a) Questionnaire cannot be used for data collection as the respondents could not understand a few terminologies (b) Additional explanations in the vernacular language needed to be provided for the farmers to get reliable answers. After scrutinizing all the above issues, it was decided to use the Structured Interview Schedule for data collection. The market for commodity futures helps the farmers to hedge against undesirable price changes at future periods. Hence, the data collection instrument was designed to capture the awareness and perception of farmers towards the futures market. Since majority use auction at the regulated markets as the primary tool for selling, it was decided to include other marketing alternatives such as local mandi, traders, cooperative societies, farmers’ associations, forward contracts, and futures markets, to obtain the data on farmers’ choice of selling.

## Ethics Statements

The data does not involve any biological or scientific experiments. Participation in the research was voluntary and the farmers were briefed clearly about the study and its significance.

## CRediT authorship contribution statement

**S. Srinivasan:** Conceptualization, Methodology, Visualization, Data curation, Formal analysis, Investigation, Writing – original draft. **M. Babu:** Conceptualization, Methodology, Writing – review & editing, Supervision, Validation. **P.S. Shabi Shimny:** Validation, Writing – review & editing, Visualization. **C. Hariharan:** Validation, Data curation, Writing – review & editing, Visualization. **J. Gayathri:** Validation, Data curation, Writing – review & editing, Visualization. **G. Indhumathi:** Validation, Writing – review & editing, Visualization.

## Declaration of Competing Interest

The authors declare that they have no known competing financial interests or personal relationships that could have appeared to influence the work reported in this paper.
